# Changing Landscape of Liver Transplantation in the Post-DAA and Contemporary ART Era

**DOI:** 10.3390/life12111755

**Published:** 2022-11-01

**Authors:** Huma Saeed, Edison J. Cano, Mohammad Qasim Khan, Zachary A. Yetmar, Byron Smith, Stacey A. Rizza, Andrew D. Badley, Maryam Mahmood, Michael D. Leise, Nathan W. Cummins

**Affiliations:** 1 Division of Infectious Diseases, Mayo Clinic, Rochester, MN 55902, USA; 2 Division of Gastroenterology and Hepatology, Mayo Clinic, Rochester, MN 55902, USA; 3 Biomedical Statistics and Informatics, Mayo Clinic, Rochester, MN 55902, USA; 4 Department of Molecular Medicine, Mayo Clinic, Rochester, MN 55902, USA

**Keywords:** liver transplantation, hepatitis C virus, HIV/AIDS, direct-acting antiviral therapy, anti-retroviral therapy

## Abstract

Combination anti-retroviral therapy has drastically improved solid organ transplantation outcomes in persons living with HIV. DAA therapy has led to the successful eradication of HCV. While recent data have suggested improvement in outcomes in HIV/HCV-coinfected liver transplant recipients, temporal trends in patient survival within pre- and post-DAA eras are yet to be elucidated. The UNOS database was utilized to identify deceased donor liver transplant recipients between 1 January 2000 and 30 September 2020 and stratify them by HIV and HCV infection status. A total of 85,730 patients met the inclusion criteria. One-year and five-year patient survival improved (93% and 80%, respectively) for all transplants performed post-2015. For HIV/HCV-coinfected recipients, survival improved significantly from 78% (pre-2015) to 92% (post-2015). Multivariate regression analyses identified advanced recipient age, Black race, diabetes mellitus and decompensated cirrhosis as risk factors associated with higher one-year mortality. Liver transplant outcomes in HIV/HCV-coinfected liver transplant recipients have significantly improved over the last quinquennium in the setting of the highly effective combination of ART and DAA therapy. The presence of HIV, HCV, HIV/HCV-coinfection and active HCV viremia at the time of transplant do not cause higher mortality risk in liver transplant recipients in the current era.

## 1. Introduction

The advent of combination antiretroviral therapy (ART) has improved the outcomes of solid organ transplantation in persons living with human immunodeficiency virus (PLWH) [1,2]. Hepatitis C virus (HCV) and HIV coinfection is common, especially in high-risk populations such as men who have sex with men (MSM) and intravenous injection drug users (IVDU), in whom prevalence rates are as high as 80% [3,4]. HIV/HCV-coinfection is associated with 23-fold higher risk of progression of hepatocellular carcinoma and 6-fold higher risk of progression to end-stage liver disease compared to HCV infection alone. As a result, there are increasing demands of liver transplantation in PLWHs [5,6,7].

Outcomes of PLWH undergoing liver transplantation are comparable to HIV-uninfected recipients, with one-year survival of 84.5% [8,9,10]. Additionally, a negative impact of post-transplant immunosuppression on HIV viral suppression has not been reported, and 87.2% of the patients remain with undetectable viral loads at one year after transplantation [10]. However, inferior survival rates are historically reported in HIV/HCV-coinfected liver transplant recipients in small series as well as large registry-based studies [11,12,13]. Hepatitis C recurrence is repeatedly described as a major contributor to mortality and graft failure [11,13]. These studies span the era of pegylated interferon and ribavirin therapy against HCV with suboptimal cure rates and frequent adverse reactions [14]. The development of direct-acting antiviral (DAA) therapy in 2011 revolutionized the landscape of HCV treatment, with improved treatment outcomes and increased organ acceptance rates from HCV-positive donors [15,16]. Furthermore, DAA therapies have also led to diminishing numbers of end-stage liver disease attributable to HCV infection [17,18]. Successful mainstream use of DAA therapy has been deployed both in pre- and post-liver transplant settings in numerous studies [19,20]. In 2019, Parrish et al. described improved patient and graft survival outcomes in a large registry-based study using the United Network for Organ Sharing (UNOS) database during the DAA era between the years 2014 and 2017 [21]. With the passing of the HIV Organ Policy Equity Act (HOPE Act) in 2013, which permitted transplantation from HIV-positive donors to HIV-positive recipients, there has been an increasing interest in evaluating liver transplantation outcomes in HIV/HCV-coinfected transplant recipients. To this end, Cotter et al. recently reported better patient and graft survival outcomes in HIV/HCV-coinfected liver transplant recipients pre-2013 and post-2013, representing pre- and post-DAA eras [22]. They also reported improved graft survival in HIV/HCV-coinfected and HCV-monoinfected patients. Notably, in parallel with the advent of DAA therapies, raltegravir was approved by the FDA in 2007 and dolutegravir in 2013, transforming the landscape of antiretroviral therapy. The temporal trends of patient survival in HIV+ liver transplant recipients through these overlapping eras remain unexplained. Concurrently, independent of DAA therapies and cART, improved surgical techniques, the development of modern immunosuppression protocols and improved patient selection likely have played an important role in improved patient survival over the past few decades. Thus, to highlight the impact of these evolving innovations and therapies on the transplant outcomes, herein we evaluate 1-year patient mortality in liver transplant recipients stratified by HIV and HCV infection status compared to their uninfected counterparts across four transplant quinquennia since 2000. Furthermore, we also evaluate risk factors associated with 1-year mortality in all liver transplant recipients undergoing transplantation after 2015 to reflect the changing trends of transplant outcomes in the current eras of DAA therapy and combination antiretroviral therapy.

## 2. Materials and Methods

### 2.1. Study Design

We conducted a registry-based, retrospective cohort study of all patients who underwent liver transplantation in the United States between 1 January 2000 and 30 September 2020. These data were gathered from the United Network for Organ Sharing/Organ Procurement and Transplantation Network (OPTN) database, which is prospectively collected from transplant programs and organ procurement organizations. HIV serostatus was recorded as positive or negative, indicated by either HIV antigen or antibody testing or HIV nucleic acid amplification testing—missing or unknown results were excluded. HCV status was recorded based on either HCV antibody or nucleic acid testing (NAT), although the latter was not available for all the transplant recipients.

### 2.2. Inclusion and Exclusion Criteria

All adult patients 18 years of age or older who underwent primary deceased donor liver transplantation after 1 January 2000 with documented pre-transplant HIV and HCV serologies were included in the study. 

Exclusion criteria included: (1) missing or unknown results of HIV and HCV serologies pre-transplant; (2) missing or incomplete 1-year follow-up data; (3) patient re-listed for transplant; (4) and living-donor liver transplant.

### 2.3. Outcomes

The primary outcome of our study was 1-year patient survival after liver transplantation, stratified by HIV and HCV infection status, before and after 2015. 

Secondary outcomes included: (1) 5-year patient survival after liver transplantation stratified by HIV and HCV infection status, before and after 2015; (2) risk factors associated with 1-year patient mortality in all liver transplants performed after 2015; (3) risk factors associated with 1-year patient mortality in HIV/HCV-coinfected liver transplant recipients after 2015.

### 2.4. Cohorts of Interest

All patients were divided into four cohorts stratified by their infection status: HIV-monoinfected, HCV-monoinfected, HIV/HCV-coinfected and HIV/HCV-uninfected recipients. We further subdivided these groups into four cohorts outlined by their transplant date: 1 January 2000–31 December 2004; 1 January 2005–31 December 2009; 1 January 2010–31 December 2014; and finally, 1 January 2015–30 September 2020. Risk factors associated with one-year patient mortality were evaluated in patients who underwent liver transplantation after 1 January 2015. The cutoff date of 1 January 2015 was used as an approximate date to represent a time whereafter DAA therapy use was incorporated into mainstream clinical practice.

### 2.5. Statistical Analysis

The categorical variables were summarized as frequencies and percentages and continuous variables were described as means ± standard deviations. Statistical analysis was performed using R. All *p*-values reported were 2-sided and a *p*-value < 0.05 was considered statistically significant. Categorical variables were compared using x^2^ or Fisher-exact testing. Kaplan–Meier curves were compared using log rank. Odds ratios (ORs) with 95% confidence intervals (CIs) for the study outcomes were estimated using logistic regression.

## 3. Results

### 3.1. Cohort Assembly

A total of 321,267 liver transplant records were found in the United Network for Organ Sharing/Organ Procurement and Transplantation Network (OPTN) database, 116,762 of which met inclusion criteria. Thirty-one thousand and thirty-two of these records were excluded due to missing HIV and HCV serology results, missing follow-up information and re-listing for liver transplantation at the time of follow-up. In the end, 85,730 patients were included in the study.

### 3.2. Patient Characteristics by Infection Status

A total of 55,232 patients were HCV- and HIV-negative, 29,272 patients had HCV-monoinfection, 244 had HIV-monoinfection and 282 patients were coinfected with HIV and HCV. The mean age was similar across all four categories (55.2 years), as was the predominance of male gender (66.1%) and White ethnicity (71.3%). In PLWH, the primary diagnosis at the time of listing was hepatocellular carcinoma (HCC) followed by alcohol-related cirrhosis (17.6% and 16%, respectively), similar to HIV- and HCV-uninfected patients (23.4% and 17.4%). However, HCC was superseded by HCV-related cirrhosis (45.5%) as the most common indication for transplantation in HCV-infected individuals. Furthermore, the prevalence of HCC was higher in HCV-monoinfected and HIV/HCV-coinfected patients (59.2% and 46.4% respectively) compared to the other two cohorts.

Other comorbidities such as diabetes mellitus, history of malignancy and portal venous thrombosis were present in 25.4%, 5.8% and 11.3% of all liver transplant recipients. A modest fraction of liver transplant recipients had decompensated cirrhosis prior to transplant, as suggested by the presence of ascites (31.4%), history of TIPS procedure (9.7%) and grade 3–4 encephalopathy (12%). The mean calculated MELD score for all transplant recipients was 22.6, although the mean MELD scores for HCV-monoinfected (19.6) and HIV/HCV-coinfected (19.9) individuals were lower than HIV/HCV-uninfected and HIV-monoinfected patients. From HIV-monoinfected and HIV/HCV-coinfected liver transplant recipients, 48.5% and 59.7% were also positive for hepatitis B core antibody (HBcAb), respectively. Detectable HBV viral load was present in 15% of HIV-monoinfected patients and 9.5% of HIV/HCV-coinfected patients at the time of transplant. In contrast, 39.9% and 12.6% of all patients with HCV and HIV/HCV-coinfection, respectively, had detectable HCV viral load at the time of transplantation.

The presence of HIV infection was associated with higher rates of transplantation from donors with Public Health Service (PHS) risk criteria for acute transmission of HIV, HBV or HCV (27.1% in HIV-infected and 31.2% in HIV/HCV-coinfected transplant recipients) compared to HCV-infected and HIV/HCV-uninfected recipients (21.5% and 19.1% respectively). Approximately 22% of all patients received a CMV-mismatched liver with likelihood of CMV mismatch being disproportionately lower in HIV+ groups as compared to HCV-monoinfected and HIV/HCV-uninfected groups. The prevalence of donor recreational drug use was comparable across all groups (40.7%), as was the prevalence of donor malignancy (3.3%).

The number of liver transplants performed in HIV-monoinfected and HIV/HCV-coinfected patients increased steadily with each subsequent quinquennium, with more than half of all liver transplants in HIV-infected patients performed after 2015 (67.7%).

PLWH spent less time on the waitlist before receiving liver transplantation (390 days, compared to 467 days across all groups). Within the cohorts, HIV/HCV-coinfected recipients had the highest 1-year and 5-year mortality (14.9%, 28.7%), followed by HCV-monoinfected, HIV-monoinfected and HIV/HCV-uninfected groups. Of note, HIV/HCV-coinfected patients were more frequently treated for acute cellular rejection (5.2%) compared to other groups, while HCV-monoinfected patients were least frequently treated for acute T-cell mediated rejection (3.3%) (Table 1).

### 3.3. Patient Characteristics by Transplant Quinquennium

When stratified by transplant quinquennium, the prevalence of HCV viremia in HCV-monoinfected and HIV/HCV-coinfected patients at the time of transplant reduced after 2015 (13.8% vs. 60% prior to 2015). Overall, 1-year mortality also reduced with each transplant quinquennium, with the lowest mortality rate observed in liver transplants conducted after 2015 (7.1%). Similarly, 5-year mortality also declined after 2015 to 5.1%, compared to 19.3% for the entire 20-year period. Treatable episodes of acute cellular rejection waned with each passing quinquennium, eventually plateauing after 2010 (Table 2).

### 3.4. Effects of Quinquennium and Infection Status on Patient Survival

**Figure 1:**
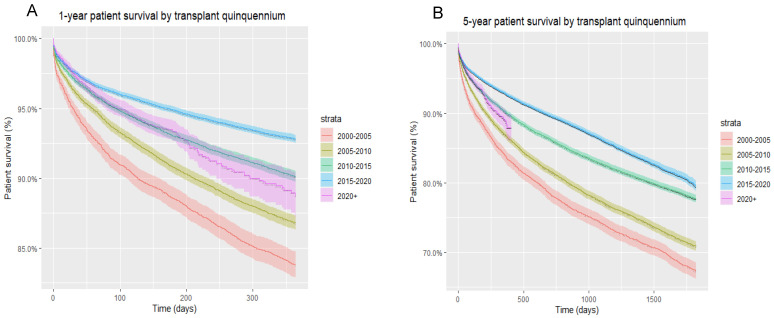
(**A**) One-year patient survival of all liver transplant recipients, by transplant quinquennium; (**B**) five-year patient survival of all liver transplant recipients, by transplant quinquennium.

Overall, one-year and five-year patient survival were highest (93% and 80%, respectively) for liver transplant recipients in the 2015–2020 quinquennium compared to prior years, irrespective of HCV and HIV status. Interestingly, with each passing quinquennium there was a progressive improvement in both one-year and five-year patient survival rates (Figure 1).

**Figure 2:**
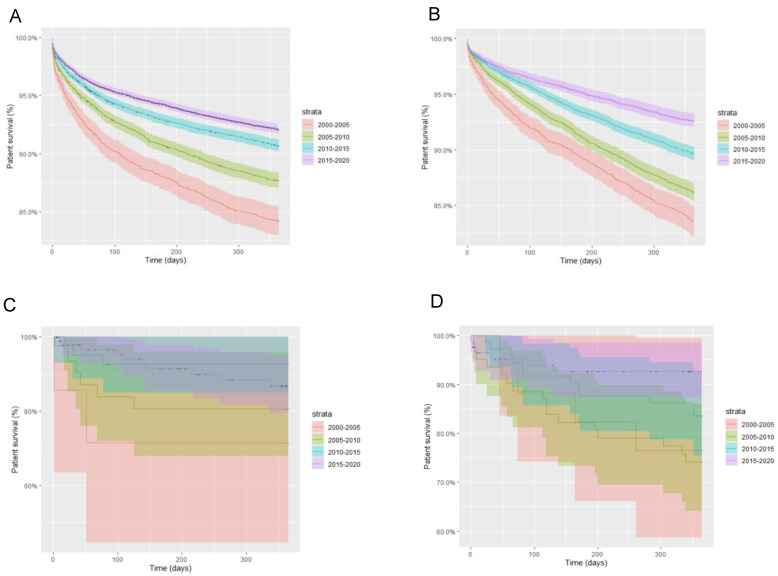
One-year patient survival stratified by liver transplant quinquennium and infection status; (**A**) HIV-negative, HCV-negative; (**B**) HIV-negative, HCV-positive; (**C**) HIV-positive, HCV-negative; (**D**) HIV-positive, HCV-positive.

Upon stratifying by HCV and HIV infection status, in each group, a similar progressive improvement in one-year patient survival was seen with each passing quinquennium. In turn, in every permutation of HCV and HIV infection status, the 2015–2020 period represented that of greatest one-year survival. Between 2000 and 2020, HIV/HCV-coinfected patients had the poorest one-year survival rates from all cohorts, followed by HIV-monoinfected patients, HCV-monoinfected patients and HCV/HIV-uninfected patients. A similar pattern was noted within these groups with respect to five-year patient survival (Figure 2).

**Figure 3:**
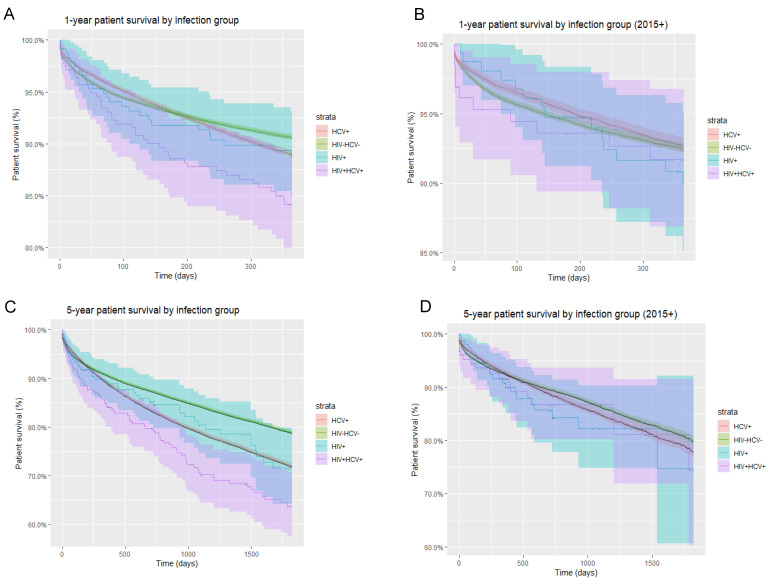
Overall and post-DAA one-year and five-year patient survival by infection group. (**A**) One-year overall patient survival stratified by infection group; (**B**) one-year patient survival stratified by infection group in the post-DAA era; (**C**) five-year overall patient survival stratified by infection group; (**D**) five-year patient survival stratified by infection group in the post-DAA era.

**Figure 4:**
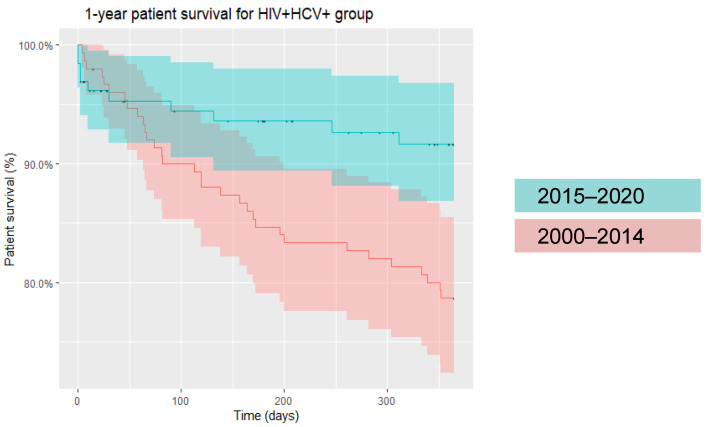
One-year patient survival in HIV/HCV-coinfected liver transplant recipients (2000–2014 and 2015-onwards).

Specifically, within the post-DAA era, i.e., 2015 onwards, as aforementioned, patients of all infection statuses demonstrated improvement in one-year patient survival. While HIV/HCV-uninfected and HCV-monoinfected patients continued to show higher one- and five-year patient survival compared to HIV-monoinfected and HIV/HCV-coinfected patients, the disparity in mortality rates between groups became less evident in the post-DAA era (Figure 3). As an example, in HIV/HCV-coinfected patients, one-year survival improved from 78% (pre-2015) to 92% (2015 onwards) (Figure 4).

### 3.5. Univariate Analysis of Factors Associated with Patient Mortality in All Liver Transplant Recipients in the Post-DAA Era 

In univariate regression analyses, advanced age (OR 1.02, CI 1.01–1.02, *p* < 0.001), Black race (OR 1.2, CI 1.05–1.37, *p* = 0.008) and underlying recipient diabetes mellitus (OR 1.3, CI 1.19–1.41, *p* < 0.001) were associated with higher mortality risk. Asian ethnicity was noted to be a protective factor (OR 0.79, CI 0.63–0.98, *p* = 0.04). Amongst donor related risk factors, inotrope use at the time of transplant (OR 1.1, 1.01–1.19, *p* = 0.02), history of hypertension (OR 1.13, CI 1.04–1.22, *p* = 0.004) and diabetes mellitus (OR 1.17, CI 1.03–1.32, *p* = 0.012) were associated with higher mortality at one year. Presence of decompensated cirrhosis as indicated by moderate ascites (OR 1.29, CI 1.16–1.43, *p* < 0.001), encephalopathy (OR 1.65, 1.49–1.83, *p* < 0.001) and history of TIPS procedure (OR 1.19, CI 1.05–1.34, *p* = 0.006) prior to transplant were associated with higher mortality. More importantly, neither presence of underlying HIV monoinfection, HCV monoinfection (OR 1.29, CI 0.71–2.15, *p* = 0.4 and OR 1, CI 0.91–1.1, *p* > 0.9 respectively) nor HIV/HCV coinfection (OR 1.15, CI 0.56–2.08, *p* = 0.7) rendered a higher mortality risk in all liver transplants performed after 2015. Furthermore, detectable HCV viral load at the time of transplant was also not associated with higher mortality risk (OR 1.03, CI 0.77–1.35, *p* = 0.9) (Table 3).

### 3.6. Predictors of Patient Mortality via Multivariate Analysis

In multivariate regression analyses, advanced age (OR 1.02, CI 1.01–1.02, *p* < 0.001), Black race (OR 1.34, CI 1.17–1.54, *p* < 0.001) and recipient diabetes mellitus (OR 1.18, CI 1.08–1.28, *p* < 0.001) remained significantly associated with higher mortality rates. Presence of grade 1–2 (OR 1.14, CI 1.03–1.27, *p* = 0.012) and grade 3–4 encephalopathy (OR 1.84, CI 1.61–2.1, *p* < 0.001) and portal vein thrombosis (OR 1.34, CI 1.21–1.49, *p* < 0.001) were independently associated with poorer outcomes in multivariate regression analysis, whereas underlying ascites was not (OR 1.07, CI 0.95–1.2, *p* = 0.3 for slight and OR 1.08, CI 0.96–1.23, *p* = 0.2 for moderate ascites) (Table 4).

## 4. Discussion

In December 2013, FDA approval of the highly effective sofosbuvir (SOF), a nucleotide analog inhibitor of HCV NS5B polymerase, changed the landscape of treatment of chronic HCV infection and led to improved mortality outcomes in HCV-infected liver transplant recipients [23]. However, survival outcomes have been historically reported to be bleaker in liver transplant recipients coinfected with HCV and HIV, with one study illustrating 1- and 5-year survival at 66.7% and 33.3%, respectively. This was predominantly due to recurrence of HCV infection following transplantation in the pre-DAA era contributing to increased incidences of graft loss and sepsis [5,11,13]. There has, however, been demonstrable improvement in recent years, with studies indicating lower incidences of graft loss in HIV/HCV-coinfected individuals in the immediate post-DAA period (2012–2015) [24].

The enactment of the HOPE Equity Act in 2013, which allowed HIV-positive patients to receive HIV-positive organs, has led to an uptrend in the number of solid organ transplants performed in these patients over time. Our study confirms this encouraging trend, with progressively higher number of liver transplantations carried out in HIV/HCV-coinfected individuals with each passing quinquennium. Most liver transplants in HIV-, HCV- and HIV/HCV-infected patients were performed in the last quinquennium, i.e., 2015–2020.

We noted high prevalence of hepatitis B infection in HIV- and HIV/HCV-coinfected liver transplant recipients, with HBcAb positivity rates as high as 48.5% and 59.7%, respectively. Amongst these, evidence of HBV viremia was present for 15% and 9.5% of recipients, respectively. However, neither HBcAb positivity nor HBV viremia conferred higher mortality risk at one-year post-liver transplant. This finding resonates with the previously published literature that shows that while presence of peri-transplant hepatitis B viremia is a predictive factor for HBV recurrence after transplant, it does not affect the overall mortality [25]. More importantly, our study concluded no significant impact of peri-transplant HCV viremia on 1-year patient mortality. This is an important factor to weigh during consideration of treatment of hepatitis C infection prior to transplant. Studies have shown improved liver function in patients with decompensated cirrhosis following successful eradication of viremia with DAA therapy, thereby leading to delisting [26]. In addition, the MELD purgatory effect, which is driven by improvement in biochemical function (MELD) without significant clinical improvement following successful treatment of hepatitis C infection, can lead to unnecessary delays in transplantation. For this reason, pre-transplant treatment is recommended only for select few patients with MELD greater than 16 [27]. Per European Association of Study of Liver (EASL) guidelines, HCV treatment prior to transplantation generally should be considered for MELD scores < 18–20 [28]. In support of these recommendations, a US study combining real world data and modelling revealed that HCV treatment in patients with MELD > 20 conferred at most 1-year survival, thus favoring transplantation in these patients with very severe disease, before HCV treatment [29]. Our finding that detectable peri-transplant HCV viral load does not impart negative consequences on one-year patient survival validates this practice of deferring initiation of antiviral treatment until after transplantation.

HIV-positive individuals were more likely to receive organs from donors that were at increased Public Health Service (PHS) risk of transmitting HBV, HCV and HIV. This was likely due to relatively lenient acceptance criteria and attitudes attributed to participation in the HOPE Act [30]. Since organ receipt from an increased PHS risk donor had no negative implications on 1-year mortality in univariate or multivariate regression modeling, this finding should offer reassurance to transplant centers and advocate for utilization of increased PHS risk donors as a means of widening the donor pool.

Across two decades, we found higher 1- and 5-year mortality rates in HIV/HCV-coinfected recipients (14.9% and 28.7%, respectively) compared to liver transplant recipients overall (9.5% and 19.3%, respectively). While HIV/HCV-coinfected liver transplant recipients had the lowest post-transplant survival in the pre-DAA era, survival increased over time with each passing quinquennium, with survival in HIV/HCV-coinfected recipients surpassing that of HIV-monoinfected individuals, although this did not reach statistical significance (Table 1). We demonstrated a similar trend of progressively improving patient survival with each passing quinquennium in HIV-monoinfected individuals, extending from the pre-DAA era through the post-DAA era. This likely represents the effect of the introduction of integrase inhibitors between 2007 and 2013 in concert with improved surgical techniques, transplant selection and immunosuppression protocols. Similar to Cotter et al., our analyses illustrate that in the current, post-DAA era, survival in HIV/HCV-coinfected liver transplant recipients has significantly improved and does not differ from other groups (Figure 3). Our data revealed 78% one-year survival in HIV/HCV-coinfected liver transplant recipients between 2000 and 2015, while survival improved significantly to 92% in the post-2015 era (Figure 4).

In the pre-DAA era, acute rejection and graft failure was a major cause of morbidity and mortality in HIV/HCV-coinfected liver transplant recipients compared to their HCV-monoinfected counterparts (39% vs. 24%, respectively, *p* = 0.01) [31]. However, a recently published study evaluating the outcomes in the same population reported no difference in graft failure in HIV/HCV-coinfected recipients compared to HCV-monoinfected ones in the current era [22]. While our study did not evaluate graft survival as an outcome, we did note an overall decreasing incidence of acute T-cell-mediated rejection from 2010 onwards across all four infection cohorts, although overall incidence of acute rejection in coinfected patients remained higher than the general population (5.2% vs. 3.8%, respectively). The improvement in trends is likely explained by the evolution of management strategies with highly effective DAA therapies and ART as well as advancements in the choice of immunosuppressants and therapeutic drug monitoring [32]. Additionally, it is encouraging to note that prior treated episodes of rejection did not influence 1-year mortality in our study.

While determining risk factors associated with 1-year patient mortality following liver transplantation in the post-DAA era, our findings were congruent with previously published reports. The presence of pre-transplant donor and recipient diabetes mellitus led to increased risk of one-year mortality. This is similar to previously reported findings of a 40% increase in the risk of death following liver transplantation in patients with pre-existing diabetes compared to their non-diabetic counterparts [33]. Furthermore, we noted an increased mortality risk in patients receiving organs from donors with diabetes. This increased mortality risk has been documented through studies evaluating graft and patient survival following renal and liver transplantation from diabetic donors [34,35]. Similarly, donor hypertension was also found to be a risk factor for 1-year mortality following liver transplantation. Various studies have reported pre- and post-transplant hypertension to be a major contributor to morbidity and mortality; however, very limited information is available regarding the effects of donor hypertension on survival outcomes [36]. Other risk factors associated with higher mortality post-liver transplant included presence of cirrhosis and end-stage liver disease pre-transplant, as suggested by high MELD scores, presence of hepatic encephalopathy, moderate ascites and portal vein thrombosis as well as history of TIPS procedure pre-transplant. Previously published studies have also reported similar findings, noting increased short-term mortality in liver transplant recipients with pre-transplant hepatic encephalopathy and higher MELD scores (>25–36) [37,38,39].

Black ethnicity was also found to be an independent risk factor contributing to 1-year mortality in the post-DAA era. This finding likely reflects the ongoing disparities in access to healthcare across ethnic groups and minorities, in combination with reduced effects and diminished bioavailability of immunosuppressants in Black patients [40,41,42]. Another possible explanation for reduced patient and graft survival in Black recipients is the effect of race-mismatched donors, with studies revealing that almost 70% of Black patients received organs from White patients [40]. In contrast, Asian ethnicity was associated with increased survival in liver transplant recipients.

The strengths of our study include a large sample size of 85,730 liver transplant recipients, allowing for a robust analysis of patients across time and various infection groups. The limitations include those inherent to the retrospective nature of our study, although missing data were very infrequently encountered and thus unlikely to have influenced results. Five-year mortality outcomes could not be assessed properly in patients that underwent liver transplantation in the years 2015–2020 due to incomplete 5-year follow-up information in patients receiving transplants in the latter half of the quinquennium. Furthermore, given that UNOS/OPTN does not collect data on HCV treatment or recurrence post-transplant, we were unable to evaluate the frequency of HCV recurrence and its influence on mortality in the post-DAA era. However, given the previously documented high sustained virologic response rates of DAA therapy in HIV/HCV-coinfected liver transplant recipients, HCV recurrence was unlikely to have contributed significantly to poor outcomes in HCV-monoinfected or HIV/HCV-coinfected patients.

## Figures and Tables

**Table 1:** Patient characteristics by infection status.

	HIV-HCV- (*n* = 55,232)	HCV+ (*n* = 29,972)	HIV+ (*n* = 244)	HIV + HCV+ (*n* = 282)	Total (*n* = 85,730)	*p*
**Age, years (mean ± SD)**	54.3 (11.7)	56.7 (7.4)	52.5 (9.5)	54.0 (8.5)	55.2 (10.4)	<0.001
**Sex**						<0.001
**Female**	21,435 (38.8%)	7524 (25.1%)	50 (20.5%)	63 (22.3%)	29,072 (33.9%)	
**Male**	33,797 (61.2%)	22,448 (74.9%)	194 (79.5%)	219 (77.7%)	56,658 (66.1%)	
**Ethnicity**						<0.001
**White**	40,258 (72.9%)	20589 (68.7%)	141 (57.8%)	158 (56.0%)	61,146 (71.3%)	
**Black**	3962 (7.2%)	3691 (12.3%)	50 (20.5%)	67 (23.8%)	7770 (9.1%)	
**Hispanic**	7570 (13.7%)	4473 (14.9%)	39 (16.0%)	47 (16.7%)	12,129 (14.1%)	
**Asian**	2625 (4.8%)	810 (2.7%)	12 (4.9%)	5 (1.8%)	3452 (4.0%)	
**Other/multiracial**	817 (1.4%)	409 (1.4%)	2 (0.8%)	5 (1.7%)	1233 (1.4%)	
**Diagnosis**						<0.001
**Hepatitis C**	886 (1.6%)	13,650 (45.5%)	7 (2.9%)	160 (56.7%)	14,703 (17.2%)	
**Hepatitis B**	1244 (2.3%)	62 (0.2%)	37 (15.2%)	2 (0.7%)	1345 (1.6%)	
**NASH**	9167 (16.6%)	259 (0.9%)	33 (13.5%)	5 (1.8%)	9464 (11.0%)	
**Alcoholic cirrhosis**	14,325 (25.9%)	548 (1.8%)	39 (16.0%)	0 (0.0%)	14,912 (17.4%)	
**Alcoholic cirrhosis and hepatitis C**	239 (0.4%)	3014 (10.1%)	1 (0.4%)	12 (4.3%)	3266 (3.8%)	
**Hepatocellular carcinoma**	8955 (16.2%)	10962 (36.6%)	43 (17.6%)	78 (27.7%)	20,038 (23.4%)	
**Autoimmune hepatitis**	2021 (3.7%)	56 (0.2%)	3 (1.2%)	0 (0.0%)	2080 (2.4%)	
**Primary biliary cirrhosis**	2239 (4.1%)	48 (0.2%)	3 (1.2%)	0 (0.0%)	2290 (2.7%)	
**Sclerosing cholangitis**	3093 (5.6%)	70 (0.2%)	8 (3.3%)	0 (0.0%)	3171 (3.7%)	
**Alpha-1-antitrypsin deficiency**	1211 (2.2%)	39 (0.1%)	5 (2.0%)	1 (0.4%)	1256 (1.5%)	
**Cryptogenic**	3767 (6.8%)	99 (0.3%)	21 (8.6%)	0 (0.0%)	3887 (4.5%)	
**Other**	5653 (10.2%)	638 (2.1%)	14 (5.7%)	15 (5.3%)	6320 (7.4%)	
**Recipient-related Factors**						
**Diabetes**	14,690 (27.4%)	6234 (21.6%)	61 (25.5%)	53 (19.2%)	21,038 (25.4%)	<0.001
**Hepatocellular carcinoma**	6143 (19.4%)	6618 (59.2%)	36 (21.1%)	65 (46.4%)	12,862 (29.8%)	<0.001
**Malignancy**	998 (3.9%)	1640 (8.2%)	6 (7.6%)	12 (7.9%)	2656 (5.8%)	<0.001
**BMI (mean SD)**	28.8 (6.1)	28.5 (5.3)	26.6 (5.3)	27.0 (5.3)	28.7 (5.8)	<0.001
**MELD at transplant**	24.3 (10.5)	19.6 (10.3)	23.7 (11.6)	19.9 (10.3)	22.6 (10.7)	<0.001
**Moderate ascites at transplant**	18,259 (33.8%)	7879 (27.2%)	69 (28.3%)	52 (18.8%)	26,259 (31.4%)	<0.001
**Grade 3–4 encephalopathy at transplant**	7381 (13.7%)	2631 (9.1%)	39 (16.0%)	19 (6.9%)	10,070 (12.0%)	<0.001
**TIPSS at transplant**	5379 (9.9%)	2771 (9.4%)	21 (8.7%)	26 (9.5%)	8197 (9.7%)	0.091
**Portal vein thrombosis at transplant**	6495 (11.9%)	3065 (10.3%)	39 (16.0%)	25 (9.0%)	9624 (11.3%)	<0.001
**Treated for acute rejection episode**	2181 (4.1%)	926 (3.3%)	10 (4.2%)	14 (5.2%)	3131 (3.8%)	<0.001
**HBcAb positive status**	5376 (10.0%)	9791 (33.9%)	115 (48.5%)	163 (59.7%)	15,445 (18.6%)	<0.001
**HB SAg positive status**	2962 (5.4%)	739 (2.5%)	64 (26.4%)	17 (6.1%)	3782 (4.5%)	<0.001
**Hepatitis B NAT positive status**	173 (3.7%)	42 (3.1%)	6 (15.0%)	4 (9.5%)	225 (3.7%)	<0.001
**Hepatitis C Ab positive status**	0 (0.0%)	29,898 (99.8%)	0 (0.0%)	278 (98.6%)	30,176 (35.2%)	<0.001
**Hepatitis C NAT positive**	0 (0.0%)	959 (39.9%)	0 (0.0%)	18 (32.7%)	977 (12.6%)	<0.001
**CMV mismatch (D+/R-)**	12,611 (23.4%)	5423 (18.8%)	19 (7.9%)	28 (10.4%)	18,081 (21.7%)	<0.001
**EBV mismatch (D+/R-)**	4424 (10.0%)	1830 (8.1%)	15 (7.2%)	14 (6.2%)	6283 (9.3%)	<0.001
**Donor-related Factors**						
**Age, years (mean SD)**	41.5 (16.8)	40.8 (15.7)	40.0 (16.0)	39.0 (14.9)	41.2 (16.4)	<0.001
**High-risk donor**	10,083 (19.1%)	5966 (21.5%)	65 (27.1%)	84 (31.2%)	16,198 (20.0%)	<0.001
**Inotrope support**	26,800 (49.2%)	14,928 (51.0%)	96 (39.8%)	151 (53.7%)	41,975 (49.8%)	<0.001
**Hypertension**	19,118 (34.9%)	9981 (33.5%)	85 (35.3%)	82 (29.6%)	29,266 (34.4%)	<0.001
**Drug use**	21,981 (40.3%)	12,216 (41.4%)	104 (43.9%)	130 (46.6%)	34,431 (40.7%)	0.003
**Cancer**	1938 (3.5%)	893 (3.0%)	10 (4.1%)	5 (1.8%)	2846 (3.3%)	<0.001
**Diabetes**	5595 (10.3%)	2896 (9.8%)	19 (7.9%)	17 (6.2%)	8527 (10.1%)	0.011
**Transplant-related factors**						
**Transplant quinquennium**						<0.001
**2000–2005**	3242 (5.9%)	2870 (9.6%)	7 (2.9%)	17 (6.0%)	6136 (7.2%)	
**2005–2010**	9371 (17.0%)	7616 (25.4%)	31 (12.7%)	62 (22.0%)	17,080 (19.9%)	
**2010–2015**	12,815 (23.2%)	9814 (32.7%)	41 (16.8%)	72 (25.5%)	22,742 (26.5%)	
**2015–2020**	23,627 (42.8%)	8485 (28.3%)	129 (52.9%)	105 (37.2%)	32,346 (37.7%)	
**2020+**	6177 (11.2%)	1187 (4.0%)	36 (14.8%)	26 (9.2%)	7426 (8.7%)	
**Number of days from listing to transplant**	226.8 (454.5)	293.2 (487.9)	203.2 (390.3)	288.0 (464.3)	250.1 (467.4)	<0.001
**Death at 1 yr**	4880 (8.8%)	3207 (10.7%)	25 (10.2%)	42 (14.9%)	8154 (9.5%)	<0.001
**Death at 5 yr**	9033 (16.4%)	7391 (24.7%)	45 (18.4%)	81 (28.7%)	16,550 (19.3%)	<0.001

**Table 2:** Patient characteristics by transplant quinquennium.

	2000–2005 (*n* = 6136)	2005–2010 (*n* = 17,080)	2010–2015 (*n* = 22,742)	2015–2020 (*n* = 32,346)	2020+ (*n* = 7426)	Total (*n* = 85,730)	*p*
**HBcAb positive **	1251 (22.0%)	3737 (23.1%)	4703 (21.4%)	4950 (15.5%)	804 (11.0%)	15445 (18.6%)	<0.001
**HBsAg positive**	339 (5.7%)	968 (5.8%)	1051 (4.7%)	1199 (3.7%)	225 (3.1%)	3782 (4.5%)	<0.001
**Hepatitis B NAT+**	0 (0.0%)	0 (0.0%)	2 (4.8%)	158 (3.9%)	65 (3.1%)	225 (3.7%)	<0.001
**Hepatitis C Ab+ **	2887 (47.1%)	7678 (45.0%)	9886 (43.5%)	8538 (26.4%)	1187 (16.0%)	30176 (35.2%)	<0.001
**Hepatitis C NAT+**	0 (0.0%)	0 (0.0%)	60 (60.0%)	689 (13.8%)	228 (8.6%)	977 (12.6%)	<0.001
**Treated acute rejection episodes**	169 (9.1%)	870 (5.1%)	752 (3.3%)	1050 (3.2%)	290 (3.9%)	3131 (3.8%)	<0.001
**Death at 1 yr**	992 (16.2%)	2251 (13.2%)	2239 (9.8%)	2295 (7.1%)	377 (5.1%)	8154 (9.5%)	<0.001
**Death at 5 yr**	2001 (32.6%)	4963 (29.1%)	5021 (22.1%)	4186 (12.9%)	379 (5.1%)	16550 (19.3%)	<0.001

**Table 3:** Univariate regression analysis.

Characteristic	OR	95% CI	*p*
**Recipient-related factors**			
Age	1.02	1.01–1.02	<0.001
Male gender	0.98	0.90–1.06	0.6
**Ethnicity**			
White	—	—	
Black	1.2	1.05–1.37	0.008
Hispanic	0.99	0.88–1.10	0.8
Asian	0.79	0.63–0.98	0.041
**Diagnosis**			
Hepatitis C	0.78	0.61–1.01	0.057
Hepatitis B	0.8	0.53–1.19	0.3
NASH	0.94	0.75–1.19	0.6
Alcoholic cirrhosis	0.6	0.48–0.76	<0.001
Alcoholic cirrhosis and hepatitis C	0.98	0.71–1.34	0.9
Hepatocellular carcinoma	0.82	0.65–1.03	0.086
Autoimmune hepatitis	0.86	0.62–1.19	0.4
Primary biliary cirrhosis	0.75	0.53–1.05	0.1
Sclerosing cholangitis	0.48	0.34–0.67	<0.001
Alpha-1-antitrypsin deficiency	0.63	0.41–0.95	0.03
Cryptogenic	1.06	0.80–1.41	0.7
Other	1	0.78–1.29	>0.9
Diabetes	1.3	1.19–1.41	<0.001
Hepatocellular carcinoma	0.95	0.87–1.04	0.3
Malignancy	0.71	0.17–2.04	0.6
Infection status			
a. HIV-/HCV-	—	—	
b. HIV-/HCV+	1	0.91–1.10	>0.9
c. HIV+/HCV-	1.29	0.71–2.15	0.4
d. HIV+/HCV+	1.15	0.56–2.08	0.7
**Donor-related Factors**			
Age	1	1.00–1.00	0.06
High-risk donor	0.93	0.85–1.01	0.1
Inotrope use	1.1	1.01–1.19	0.02
Hypertension	1.13	1.04–1.22	0.004
Drug use	0.88	0.81–0.95	0.001
Cancer	0.96	0.76–1.19	0.7
Diabetes	1.17	1.03–1.32	0.012
History of documented infection	0.97	0.89–1.06	0.5
**Transplantation-related factors**			
MELD score	1.01	1.01–1.02	<0.001
Moderate and severe ascites *	1.17	1.08–1.27	<0.001
Moderate encephalopathy **	1.65	1.49–1.83	<0.001
TIPSS at the time of transplant	1.19	1.05–1.34	0.006
Portal vein thrombosis	1.45	1.31, 1.60	<0.001
Treated episodes of acute rejection	1.09	0.87–1.33	0.4
ABO incompatibility	0.69	0.45–1.02	0.076
HBcAb positive	0.99	0.89–1.11	>0.9
HBsAg positive	0.96	0.77–1.18	0.7
HBsAb positive	1.05	0.96–1.15	0.3
HBVNAT positive	0.61	0.29–1.12	0.15
HCV Ab positive	1.01	0.92–1.10	>0.9
HCV NAT positive	1.03	0.77–1.35	0.9
CMV mismatch	1.08	0.94–1.23	0.3
EBV mismatch	1.58	0.84–3.38	0.2

*: Moderate and severe ascites were defined by grade 2 and grade 3 ascites. **: Moderate and severe encephalopathy were described by grade 2–4 hepatic encephalopathy.

**Table 4:** Multivariate regression analyses—risk factors associated with one-year mortality in liver transplant recipients in the post-DAA era.

Characteristic	OR	95% CI	*p*
Age	1.02	1.01–1.02	<0.001
Black race	1.34	1.17–1.54	<0.001
Recipient diabetes	1.18	1.08–1.28	<0.001
Donor hypertension	1.05	0.96–1.15	0.3
Donor diabetes	1.12	0.99–1.27	0.077
Ascites at time of transplant			
● Slight	1.07	0.95–1.20	0.3
● Moderate	1.08	0.96–1.23	0.2
Encephalopathy at transplant			
● grade 1–2	1.14	1.03–1.27	0.012
● grade 3–4	1.84	1.61–2.10	<0.001
TIPSS at transplant	1.1	0.97–1.24	0.14
Portal vein thrombosis	1.34	1.21–1.49	<0.001

## Data Availability

Access to the UNOS database is available upon request through the UNOS portal.

## Abbreviations

ART:	Anti-Retroviral Therapy
CI:	Confidence Interval
CMV:	Cytomegalovirus
DAA:	Direct Acting Antivirals
EASL:	European Association for the Study of the Liver
HBcAb:	Hepatitis B Core Antibody
HBV:	Hepatitis B Virus
HCV:	Hepatitis C Virus
HIV:	Human Immunodeficiency Virus
HOPE:	HIV Organ Policy Equity Act
MELD:	Model for End-Stage Liver Disease
OPTN:	Organ Procurement and Transplantation Network
OR:	Odds Ratio
PLWH:	Patients living with HIV
SOF:	Sofosbuvir
TIPS:	Trans-jugular Intrahepatic Porto-systemic Shunt
UNOS:	United Network for Organ Sharing
